# Differences in the effectiveness of single, dual, and triple inhaled corticosteroid therapy for reducing future risk of severe asthma exacerbation: A systematic review and network meta-analysis

**DOI:** 10.1016/j.heliyon.2024.e31186

**Published:** 2024-05-16

**Authors:** Akira Yamasaki, Katsuyuki Tomita, Genki Inui, Ryota Okazaki, Tomoya Harada

**Affiliations:** a Division of Respiratory Medicine and Rheumatology, Department of Multidisciplinary Internal Medicine, Faculty of Medicine, Tottori University, Tottori, Japan; b Department of Respiratory Medicine, National Hospital Organization Yonago Medical Center, Tottori, Japan

**Keywords:** Adult, Asthma, Exacerbations, Inhaled corticosteroids, Network meta-analysis

## Abstract

**Importance:**

The effectiveness of different combinations of inhaled corticosteroid (ICS) therapies in reducing severe exacerbations of adult asthma remains unclear.

**Objective:**

This network meta-analysis (NMA) extensively evaluated the treatment effects of single ICS; dual ICS i.e., ICS/long-acting β2-adrenergic agonists (LABA); ICS/LABA as single maintenance and reliever therapy (SMART); and triple ICS, i.e., ICS/LABA/long-acting muscarinic antagonists (LAMA) in preventing severe asthma exacerbations.

**Data sources:**

A systematic search of English databases, including PubMed and Web of Science, was conducted until December 31, 2022, using PRISMA-NMA.

**Study selection:**

Using the PICOS criteria, the questions for this study were carefully selected so that the correct keywords could be identified.

**Data extraction and synthesis:**

A pairwise meta-analysis was used to select trials based on the criteria for minimizing heterogeneity (I^2^). Subsequently, the “BUGSnet” package of R software was used to perform a Bayesian network meta-analysis.

**Main outcome measures:**

The main outcome measures were risk rate and annualized rate ratio of severe asthma exacerbations.

**Results:**

This review included 56 randomized control trials (RCTs; n = 78,171 patients). As the pairwise meta-analysis demonstrated that the annualized rate ratio of severe asthma exacerbation had moderate heterogeneity, we analyzed the risk rate of severe asthma exacerbation using a network meta-analysis. In terms of direct/indirect comparisons with non-ICS, single ICS, dual ICS, SMART, and triple ICS reduced severe asthma exacerbations by 34 %, 47 %, 58 %, and 57 %, respectively. SMART and triple ICS showed high effectiveness in reducing severe exacerbations.

**Conclusion:**

AND RELEVANCE: SMART and triple ICS were ranked higher in effectiveness in reducing severe asthma exacerbations in comparison with other therapies, indicating that these are the most effective treatments for reducing the future risk of severe asthma exacerbations.

## 1 Introduction

Asthma is a chronic inflammatory condition that causes flare-ups (exacerbations) and imposes a heavy burden on patients. Inhaled corticosteroids (ICSs) are a cornerstone of asthma maintenance therapy [1]. ICSs exert profound anti-inflammatory effects through the activation/repression of multiple transcriptional networks [2]. Multiple studies have shown that treatment with ICS reduces symptoms, improves lung function, controls asthma, and prevents exacerbations [[3], [4], [5]].

The first synthetic corticosteroid used to treat asthma was beclomethasone dipropionate (BDP), which was initially made available for use in clinical settings as an inhalation agent in 1972 [6]. Subsequently, budesonide was demonstrated to be as effective as the new ICS when administered twice daily [7]. A previous meta-analysis demonstrated that use of a single ICS reduced the relative risk of exacerbations by approximately 55 % [8]. In the second stage, ICS/long-acting β2-adrenergic agonists (LABA) combination therapy was approved in 2000, considering both current asthma control and future risk of exacerbations [9]. These dual ICS therapies reduced exacerbation rates more than did single ICS therapy [10]. In the third stage, ICS/LABA as a single maintenance and reliever therapy (SMART) was launched in 2005 and has resulted in a significant reduction in severe asthma exacerbations in adults and adolescents [[11], [12], [13], [14]]. Compared to increasing or maintaining the dosage of ICS/LABA maintenance plus short-acting β2-adrenergic agonists (SABA) relief therapy, switching to the SMART regimen was associated with an extension of the period to the first severe exacerbation in patients with poor asthma management [15]. In the fourth stage, long-acting muscarinic antagonist (LAMA) therapy added-on to the ICS/LABA combination (triple ICS) was approved in 2017 and was shown to be efficacious in reducing exacerbations [16] and improved management of asthma control in patients with uncontrolled asthma [17].

Meta-analysis is a statistical approach that can be used to synthesize related papers included in systematic reviews. A network meta-analysis (NMA) is a statistical method that can be used to compare various therapies by combining direct and indirect evidence across a network of studies [18,19]. The benefits of different inhaled ICS therapies in reducing severe asthma exacerbations have been described in recent years through numerous systematic evaluations that incorporated NMA methodologies [[20], [21], [22], [23], [24], [25], [26], [27], [28], [29], [30]]. However, trials evaluating various therapies have adopted different strategies to account for clinical heterogeneity. If any inequality in the distribution of effect modifiers exist across trials, the outcomes regarding treatment with ICS to prevent severe asthma exacerbations will be biased [31].

In this study, we used traditional pairwise meta-analysis and multivariate random-effects Bayesian NMA for direct and indirect comparisons between combined inhaled therapies, respectively.

## 2 Methods and materials

### 2.1 Search strategy and study selection

Using the keywords “asthma,” “exacerbations,” “adult,” “randomized,” and “inhaled-corticosteroids” ([beclomethosone or budesonide or ciclesonide or fluticasone or mometasone] and [acidinum or indacaterol or ododaterol or salmeterol or vilanterol]), up to December 31st, 2022, we sought to identify all randomized control trials (RCTs) investigating prevention of asthma exacerbations.

The PICOS framework, which considers participants, interventions, comparators, outcomes, and research designs, was used to guide study selection [32].

The following were the inclusion requirements:

(1) Participants: adolescent (≥12 years old) and adult patients with asthma
(2) Intervention: Inhaled ICS interventions (e.g., single ICS, ICS/LANA, SMART, and ICS/LABA/LAMA) administrated for 8 weeks from baseline to follow-up assessment
(3) Comparator groups: control groups (e.g., non-ICS and stand-inhaled ICS).
(4) Outcomes: relative rate and annualized rate ratio of severe asthma exacerbations
(5) Study design: RCT reports.

Severe exacerbations are usually defined as visits to the emergency room (ER), hospital admission (hospitalization), or use of oral corticosteroids (OCS) for at least 3 days [33]. As the prevalence of ER visits and hospitalization are as low as 10 % of the prevalence of OCS use [34], ER visits alone, hospitalization alone, and ER visits/hospitalization were excluded as severe asthma exacerbations in this study. Non-ICS therapy included single therapy with theophylline and SABA.

The exclusion criteria were as follows:

(1) Participants: Pediatric asthma and chronic obstructive pulmonary disease
(2) Intervention: Anti-interleukin therapy
(3) Comparator groups: anti-interleukin therapy
(4) Outcomes: Mild-to-moderate asthma exacerbations
(5) Study design: Grey literature, abstracts, conference proceedings, posters, or protocols; full text unavailable

### 2.2 Data extraction

Two reviewers (AY and KT) independently selected eligible studies obtained from the literature review and extracted the following information: year of publication, sample size, length of follow-up, mean or median age, sex, smoking status, baseline forced expiratory volume in 1 s (FEV1), asthma control questionnaire (ACQ) scores, descriptions of interventions and comparators, and treatment category. In this study, the ICS dose was considered as low dose (BDP equivalent ≤400 mcg BDP), medium dose (BDP equivalent 400–1,000 mcg BDP), and high dose (BDP equivalent ≥1,000 mcg BDP) [35].

### 2.3 Assessment of network assumed

Network transitivity (potential modifiers of treatment effects are distributed similarly across direct trials), consistency (estimates of consistency between indirect and direct effects), and homogeneity (treatment effect interpretation should be homogeneous throughout the trial) were satisfied to ensure the validity of performing an NMA.

### 2.4 Risk of bias assessment

Using RoB2, a modified Cochrane risk-of-bias technique for RCTs, two reviewers (AY and KT) independently evaluated the risk-of-bias [5]. The response options on the tool were “definitely or probably yes” (a low risk-of-bias) and “definitely or probably no” (a high risk-of-bias). The items comprised five parts: randomization procedure, divergence from intended interventions, lack of outcome data, assessment of the outcome, and choice of the reported result.

### 2.5 Statistical analysis

This study follows the Preferred Reporting Items for Systematic Reviews and Meta-Analyses (PRISMA) extension statement for the meta-analysis guidelines. First, a frequentist pairwise meta-analysis of accumulated data was performed to explore estimates of heterogeneity among the studies, and a random-effects model was used. The metrics of analysis for the primary outcome were severe asthma exacerbations, evaluated as the incidence risk rate and the annualized rate ratio of severe asthma exacerbations. Statistical heterogeneity (I^2^) is a statistic that indicates the percentage of variance in a pairwise meta-analysis [36,37]. All pairwise meta-analyses were performed using the “meta” package of R software version 4.3.0 (R Foundation for Statistical Computing, Vienna, Austria).

NMA was conducted on estimated event rates, which are absolute measures of effect, rate ratios (RRs), which are relative measures of effect, and the likelihood rank of each treatment's impact on reducing severe exacerbations. Three chains of 10,000 burn-in iterations were employed in the final estimation algorithms, and 500,000 estimation iterations were used for the analysis. When the correct mixing of the three chains was shown in the trace plots from the Markov Chain Monte Carlo analysis, a particular chain-assessed model converged. The “BUGSnet” program (MRC Biostatistics Unit, Cambridge University, UK) and the JAGS program in R software (R Foundation for Statistical Computing) were used to conduct all NMAs [38].

To rate treatments in accordance with the outcomes, a Surface Under the Cumulative Ranking Curve (SUCRA) analysis was performed. The inconsistency model also assessed the incoherence assumption (i.e., the statistical discrepancy between direct and indirect evidence in a closed loop). Deviance information criteria (DIC) were used to assess model choice and goodness-of-fit. The residual deviance of the two models was compared to determine their suitability, and a close match, defined as <3 of difference in DIC, between them was deemed sufficient. Through visual inspection, convergence was evaluated using the Gelman–Robin–Brooks diagnostic, and a potential scale reduction factor (PSRF) value of 1.05 was considered as a sign that the simulation was accurate.

## 3 Results

### 3.1 Data selection

**Fig. 1:**
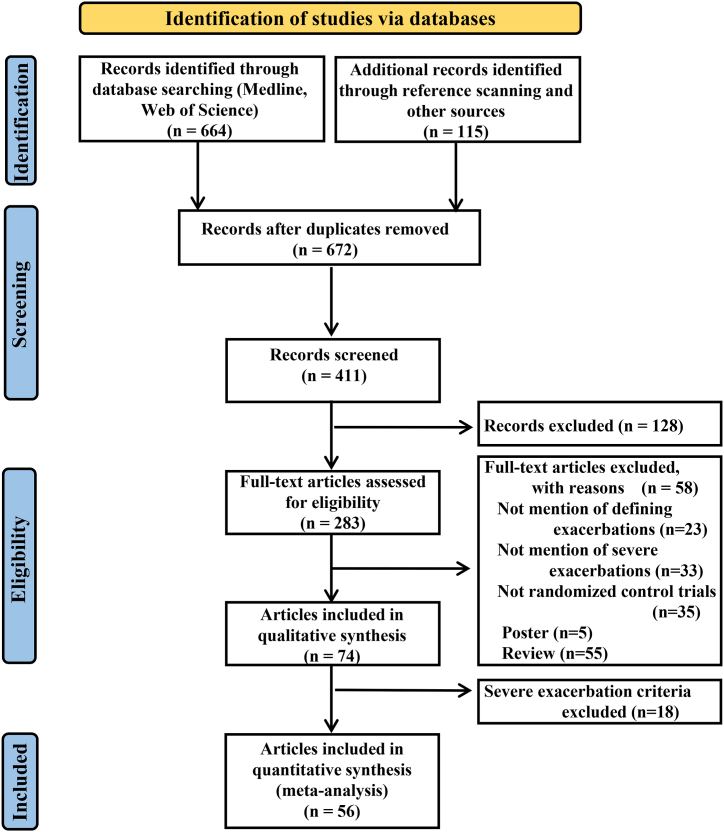
Preferred Reporting Items for Systematic Review and Meta-analysis (PRISMA) flow chart of study selection for the pairwise and network meta-analysis.

A total of 672 pertinent studies conducted from 1990 to 2022 were identified through a systematic literature search. After examining the research demographics, outcomes, population, design, intervention, and follow-up duration, 283 papers were selected for full-text evaluation, of which 56 were deemed to fit the inclusion criteria. One hundred and ten papers were excluded for the following reasons: inappropriate study population and same-category intervention (Fig. 1). Through this process, 43 studies [[39], [40], [41], [42], [43], [44], [45], [46], [47], [48], [49], [50], [51], [52], [53], [54], [55], [56], [57], [58], [59], [60], [61], [62], [63], [64], [65], [66], [67], [68], [69], [70], [71], [72], [73], [74], [75], [76], [77], [78], [79], [80], [81]] with 63,097 patients and 25 studies [12,62,[69], [70], [71],[74], [75], [76], [77], [78],[81], [82], [83], [84], [85], [86], [87], [88], [89], [90], [91], [92], [93], [94]] with 51,994 patients were included in the pairwise analysis to assess the incidence and annualized rate ratios, respectively.

### 3.2 Risk-of-Bias

**Fig. 2:**
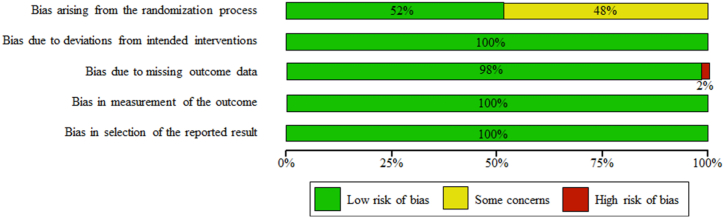
Assessment of the risk-of-bias in the included studies using Bos2.

The risk-of-bias assessment in the RCTs is shown in Fig. 2. This study included only studies with a low risk-of-bias.

### 3.3 Pairwise meta-analysis on the effectiveness of ICS therapies to reduce severe asthma exacerbations

**Fig. 3:**
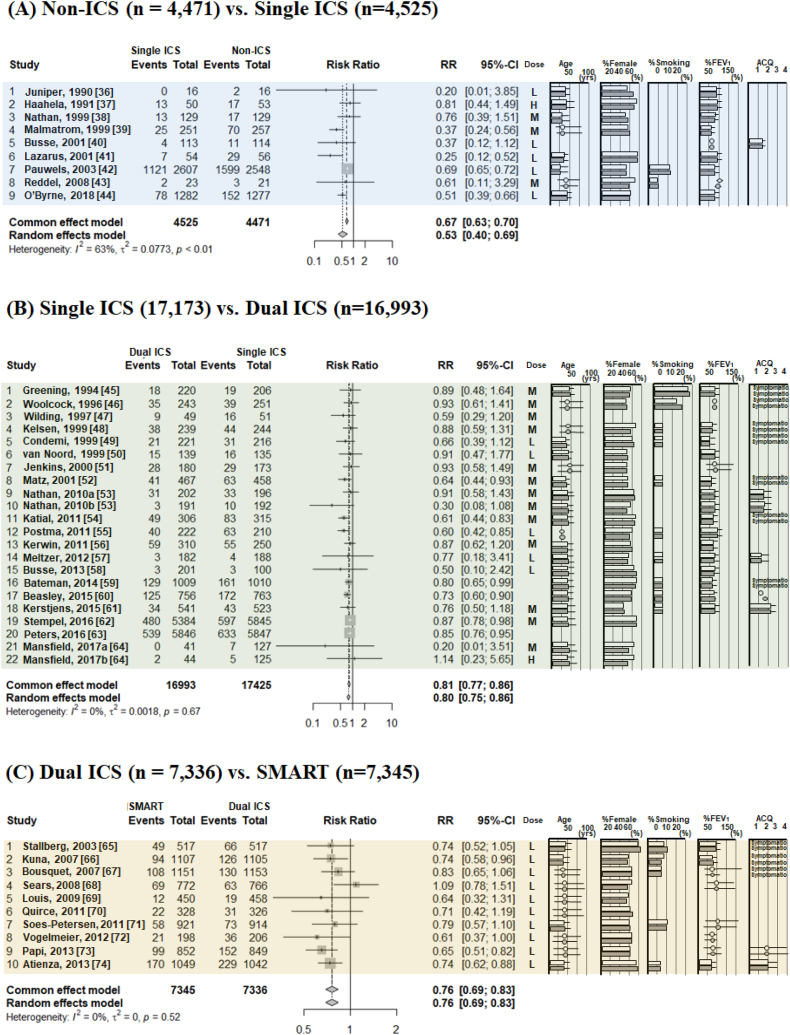

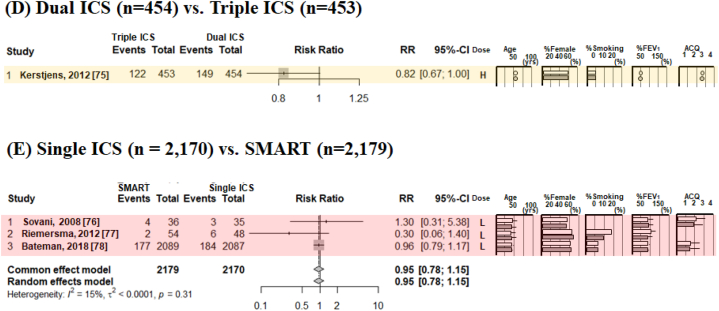
The incidence risk ratio of severe asthma exacerbations, analyzed by pairwise meta-analysis in comparisons with non-ICS versus single ICS (A), single ICS versus dual ICS (B), dual ICS versus SMART (C), dual ICS versus triple ICS (D), and single ICS versus SMART (E). Patient characteristics included mean or median age, sex (as percent female), smoking status, lung function (as percent predicted forced expiratory volume in 1 s, and asthma control status as per ACQ. ICS doses were defined as low dose (L; BDP equivalent ≤400 mcg BDP), medium dose (M; BDP equivalent 400–1000 mcg BDP), and high dose (H; BDP equivalent ≥1000 mcg BDP) [35]. ***Abbreviations:*** ACQ, asthma control questionnaire; BDP; Beclomethasone dipropionate; ICS, inhaled corticosteroid; SMART, ICS/LABA as a single maintenance and reliever; LABA, long-acting β2-adrenergic agonist.

**Fig. 4:**
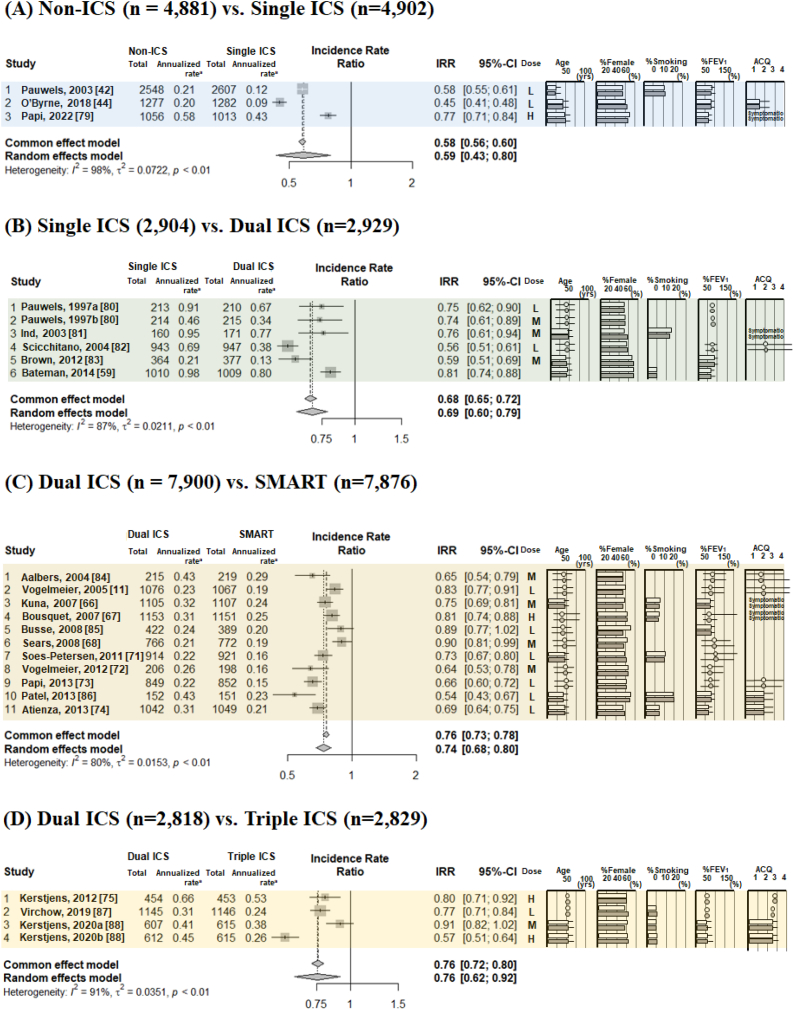

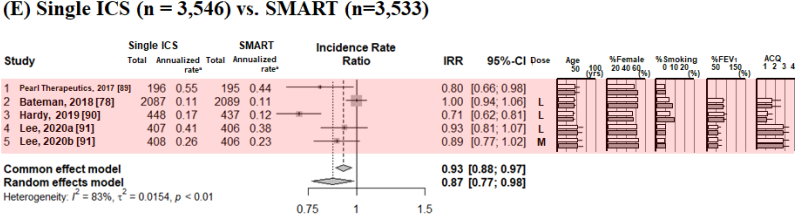
The annualized rate ratio of severe asthma exacerbations, analyzed by pairwise meta-analysis in comparisons with non-ICS versus single ICS (A), single ICS versus dual ICS (B), dual ICS versus SMART (C), dual ICS versus triple ICS (D), and single ICS versus SMART (E). The mean annualized rate ratio of severe asthma exacerbations indicated the mean number of exacerbations per patient per year (the total number of exacerbations in a group divided by the total follow-up time of the group). Patient characteristics included mean or median age, sex (as percent female), smoking status, lung function (as percent predicted forced expiratory volume in 1 s, and asthma control status as ACQ. ICS doses were defined as low dose (L; BDP equivalent ≤400 mcg BDP), medium dose (M; BDP equivalent 400–1000 mcg BDP), and high dose (H; BDP equivalent ≥1000 mcg BDP) [35]. ***Abbreviations:*** ACQ, asthma control questionnaire; BDP; Beclomethasone dipropionate; ICS, inhaled corticosteroid; SMART, ICS/LABA as a single maintenance and relief therapy; LABA, long-acting β2-adrenergic agonist.

Fig. 3, Fig. 4 show typical pairwise meta-analyses along with forest plots illustrating the trial results from these studies. The absolute risk ratio (RR) of severe asthma exacerbation was 0.25 (95 % confidence interval [CI] 0.18–0.39) for non-ICS, 0.15 (95%CI 0.12–0.18) for single ICS, 0.12 (95%CI 0.10–0.15) for dual ICS, 0.10 (95%CI 0.07–0.84) for SMART, and 0.30 (95%CI 0.04–0.84) for triple ICS. In direct comparisons between ICS therapies on the RR for severe asthma exacerbations (Fig. 3A–E), compared to non-ICS, single ICS reduced the risk by 47 % (RR 0.53, 95%CI 0.40–0.69); compared to single ICS, dual ICS reduced the irk by 21 % (RR 0.79, 95%CI 0.71–0.88); compared to dual ICS, SMART reduced the risk by 24 % (RR 0.76, 95%CI 0.69–0.83); compared to dual ICS, triple ICS reduced the risk by 18 % (RR 0.82, 95%CI 0.67–1.00); and compared to single ICS, SMART reduced the risk by 5 % (RR 0.95, 95%CI 0.78–1.15). Direct comparisons of non-ICSs and single ICS for analyzing the RR of severe asthma exacerbations were identified as interventions with high I^2^ values (63 %).

For the annualized rate ratio (ARR), the risk was reduced by 41 % by single ICS as compared to non-ICS (ARR 0.59, 95%CI 0.43–0.80), by 34 % dual ICS compared to single ICS (ARR 0.66, 95%CI 0.58–0.76), by 26 % by SMART compared to dual ICS (ARR 0.74, 95%CI 0.68–0.80), by 24 % by triple ICS compared to dual ICS (ARR 0.76, 95%CI 0.62–0.92), and by 13 % by SMART compared to single ICS (ARR 0.87, 95%CI 0.77–0.98).

Direct comparisons of all sets, non-ICS vs. single ICS, single ICS vs. dual ICS, dual ICS vs. SMART, and single ICS vs. SMART for analyzing the ARR of severe asthma exacerbations were identified as interventions with high I^2^ values (75–98 %) (Figs. 4A–４E).

### 3.4 NMAs on the effectiveness of ICS therapies to reduce severe asthma exacerbations

When five studies [39,[42], [43], [44],^47^] were excluded due to the small numbers of eligible patients for re-analyzing the direct comparison between non-ICS and single ICS by means of pairwise meta-analysis, heterogeneity became low grade (I^2^ 41 %). NMA was conducted using 38 studies [40,41,45,[47], [48], [49], [50], [51], [52], [53], [54], [55], [56], [57], [58], [59], [60], [61], [62], [63], [64], [65], [66], [67], [68], [69], [70], [71], [72], [73], [74], [75], [76], [77], [78], [79], [80], [81]] with 62,176 patients to analyze the RR for severe asthma exacerbations. However, because the pairwise meta-analysis for ARR was not guaranteed to be homogenous, we did not conduct an NMA.

**Fig. 5:**
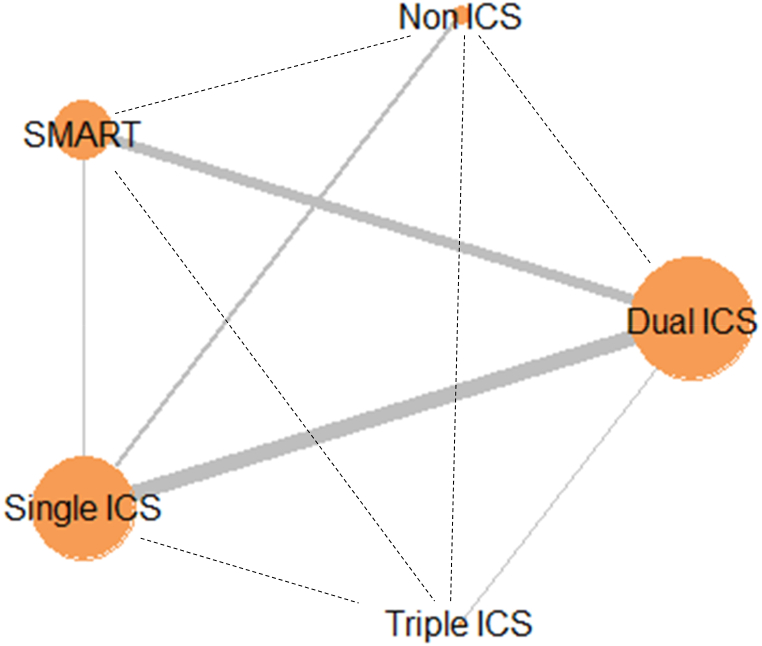
Network plot for incidence risk rate of severe asthma exacerbations, analyzed by network meta-analysis. Solid and dotted line denotes direct and indirect comparison between each inhaler therapy, respectively. ***Abbreviations:*** ICS, inhaled corticosteroid; SMART, ICS/LABA as a single maintenance and relief therapy; LABA, long-acting β2-adrenergic agonist.

Fig. 5 shows a network plot of ICS therapies according to the RR for severe asthma exacerbation. Both fixed-effects and random-effects model fits were examined. According to a visual comparison of the DIC values and leverage plots, the random-effects model was suggested efficiency compared with the fixed-effects model because the DIC value is lower and the leverage plot contains fewer outliers (**Supplementary 1A**). A Gelman–Rubin–Brooks plot was used to evaluate the convergence, which revealed that the simulations were valid because the PSRF was <1.05 (**Supplementary 1B**, Gelman convergence plot). A league table summarizing the results of the indirect comparisons is presented in Table 1.

**Table 1:**
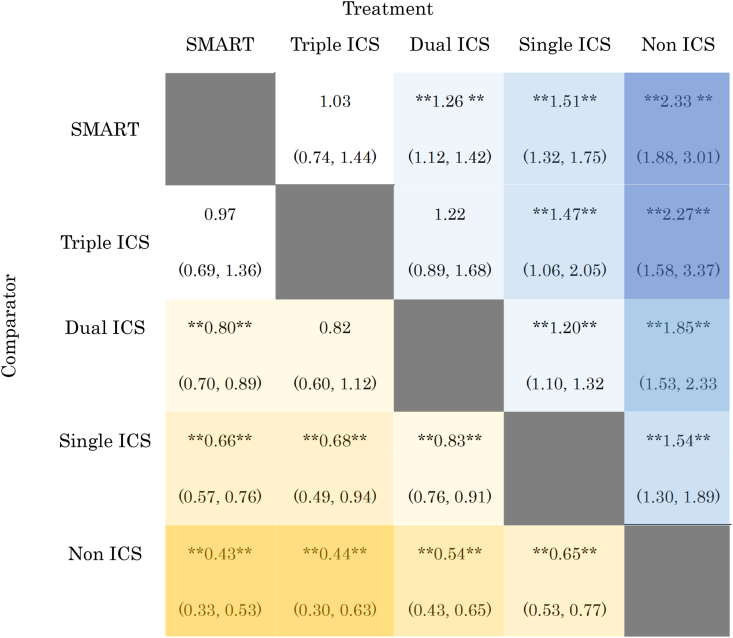
League table summarizing the results of the indirect comparisons of different ICS combination treatments for preventing severe asthma exacerbations.

The numbers in the cells denote the mean incidence risk rate (95 % confidence interval).

** **: P value < 0.05.

***Abbreviations***: ICS, inhaled corticosteroid; SMART, ICS/LABA as a single maintenance and relief therapy; LABA, long-acting β2-adrenergic agonist.

Based on the random effects, the first Bayesian indirect comparison was performed. In direct comparisons between ICS therapies on RR of severe asthma exacerbations, single ICS reduced the risk by 35 % compared to non-ICS (RR 0.65, 95%CI 0.53–0.77), dual ICS reduced it by 17 % compared to single ICS (RR 0.83, 95%CI 0.76–0.91), SMART reduced it by 20 % compared to dual ICS (RR 0.80, 95%CI 0.70–0.89), triple ICS reduced it by 18 % compared to dual ICS (RR 0.82, 95%CI 0.60–1.12), and SMART reduced it by 34 % compared to single ICS (RR 0.66, 95%CI 0.57–0.76). Data from all four studies were used in the NMA model to produce summary estimates comparable to those from the unadjusted NMA. In indirect comparisons between ICS therapies on RR of severe asthma exacerbations, dual ICS reduced the risk by 46 % compared to non-ICS (RR 0.54, 95%CI 0.43–0.65), SMART reduced it by 57 % compared to non-ICS (RR 0.43, 95%CI 0.33–0.53), triple ICS reduced it by 56 % compared to non-ICS (RR 0.44, 95%CI 0.30–0.63), triple ICS reduced it by 32 % compared to single ICS (RR 0.68, 95%CI 0.49–0.94), and SMART reduced it by 3 % compared to triple ICS (RR 0.97, 95%CI 0.69–1.36).

Both SMART and triple ICS appeared to be the most advantageous therapies with regard to reducing the RR of severe asthma exacerbations, as seen in the SUCRA plot (**Supplementary 2**), where the curve is continuously above those of the other treatments. SMART was the best-ranked treatment (SUCRA 90 %), followed by triple ICS (SUCRA 74 %) and dual ICS (SUCRA 53 %). Single ICS treatment was the lowest-ranked group for reducing the risk of future severe asthma exacerbations (SUCRA 25 %).

The leverage plots and comparison of the DIC values revealed no discernible differences between the consistent and inconsistent models (**Supplementary 3A and 3B**). We concluded that the evidence was insufficient to confirm that the network was inconsistent.

## 4 Discussion

Asthma treatment has been developed to improve asthma control and to prevent future exacerbations, with the launch of ICS being a turning point. RCT results have demonstrated the effectiveness of inhaler therapy in reducing asthma exacerbations; however, no report to date had indicated which inhaler therapy is most effective in reducing asthma exacerbations. In this NMA, by using indirect comparisons with non-ICS, we demonstrated that triple ICS (ICS/LABA/LAMA) and SMART reduced the risk of future severe asthma exacerbations by approximately 60 %.

Our findings showed an absolute incidence rate of 0.28 of asthma exacerbations, which was in line with past studies that revealed that 18.8–22.0 % of patients with mild asthma experienced at least one severe exacerbation in the previous year [95,96].

Considering the results of our NMA, we found that SMART was the highest-ranked treatment for preventing future risks, followed by triple ICS (**Supplementary 2**). Our findings are in line with earlier findings that triple therapy, as compared with dual therapy using NMA, was associated with a lower risk of severe exacerbation [27]. Because a trial of triple ICS has been reported for evaluating the RR of severe asthma exacerbations, our results were vague in determining which of SMART and triple ICS were better at preventing severe asthma exacerbations. Future studies are required to determine their comparative effectiveness for reducing severe asthma exacerbation.

In the meta-analysis, heterogeneity was defined as the difference in results between studies. The I^2^ statistic indicates the proportion of variation between studies that results from heterogeneity as opposed to that which is due to chance [97,98]. We highlighted the causes of baseline imbalances that were associated with severe exacerbations in each RCT. When study heterogeneity significantly exceeded the within-study sampling and measurement variability, the I^2^ statistic, the most widely used data-based measure of study heterogeneity, approached a maximum of 1 [99]. Differences in data or study designs may be responsible for the heterogeneity, such as different study target populations or targeted effects, survey administration and recruitment procedures, measurement tools, intervention doses, timing of outcome measurements, and analytical techniques, such as covariate adjustments. To infer causal effects, RCTs aim to minimize bias and establish groups with similar baselines. The validity of treatment effects in pairwise meta-analyses may be jeopardized by a baseline imbalance. A meta-analysis of baseline values may reveal baseline bias for significant prognostic factors, which could jeopardize the reliability of the findings. The fundamental assumption is that no imbalance exists in the distribution of effect modifiers, which are study and patient factors related to treatment effects, across various types of direct treatment comparisons. This provides a foundation for the validity of NMAs of RCTs [100]. In our study, the heterogeneity of NMA was ensured by arranging homogeneous trials in a pairwise meta-analysis by reducing the number of trials. Instead, our findings demonstrated no contradiction between direct and indirect comparisons in the majority of Bayesian-based therapies.

Because of their infrequent assessment in the included studies, moderating factors, such as recent exacerbations, lung function, and asthma control status, could not be considered in our investigation. Asthma is a heterogeneous disease with varying degrees of airway inflammation and variable responses to ICS treatment. Variations in characteristics of asthma, such as recent exacerbations [101,102], lung function [103], and asthma control status [104,105], contribute to the prevalence of severe asthma exacerbations. However, NMA permits estimation of all pairwise treatment comparisons using both direct and indirect information from a collection of RCTs. Indirect estimates from am NMA may be preferred when they can eliminate trial-specific biases that are occasionally difficult to detect in a head-to-head meta-analysis [106,107].

In conclusion, during approximately 50 years since ICS was launched, these therapies have been contributing to reducing the risk of severe asthma exacerbations: single ICS reduced this risk by 34 %, dual ICS by 47 %, SMART by 58 %, and triple ICS by 57 %. Future studies are needed to determine which inhaled ICS therapies, particularly which of SMART or triple ICS, are beneficial for reducing severe exacerbations, depending on the phenotype of asthma.

## Key points

### Question

Which combination of inhaled corticosteroid (ICS) therapies performs best in reducing severe exacerbations of adult asthma?

### Findings

Compared directly or indirectly to non-ICS treatment, single ICS, dual ICS, SMART, and triple ICS reduced severe asthma exacerbations by 34 %, 47 %, 58 %, and 57 %, respectively.

### Meaning

SMART and triple ICS are the most effective treatments for reducing the risk of severe asthma exacerbations in future.

## Data availability statement

The datasets analyzed in the current study are available from the corresponding author on reasonable request.

## Funding

This research received no external funding.

## CRediT authorship contribution statement

**Akira Yamasaki:** Writing – review & editing, Writing – original draft, Formal analysis, Data curation, Conceptualization. **Katsuyuki Tomita:** Writing – review & editing, Writing – original draft, Formal analysis, Data curation, Conceptualization. **Genki Inui:** Data curation. **Ryota Okazaki:** Data curation. **Tomoya Harada:** Data curation.

## Declaration of competing interest

The authors declare that they have no known competing financial interests or personal relationships that could have appeared to influence the work reported in this paper.
