# Supplementary figures and images for: Differences in the effectiveness of single, dual, and triple inhaled corticosteroid therapy for reducing future risk of severe asthma exacerbation: A systematic review and network meta-analysis
Source: Heliyon. 2024 May 16;10(12):e31186. doi: 10.1016/j.heliyon.2024.e31186 (PMC11252599; doi:10.1016/j.heliyon.2024.e31186)

## Slide 1
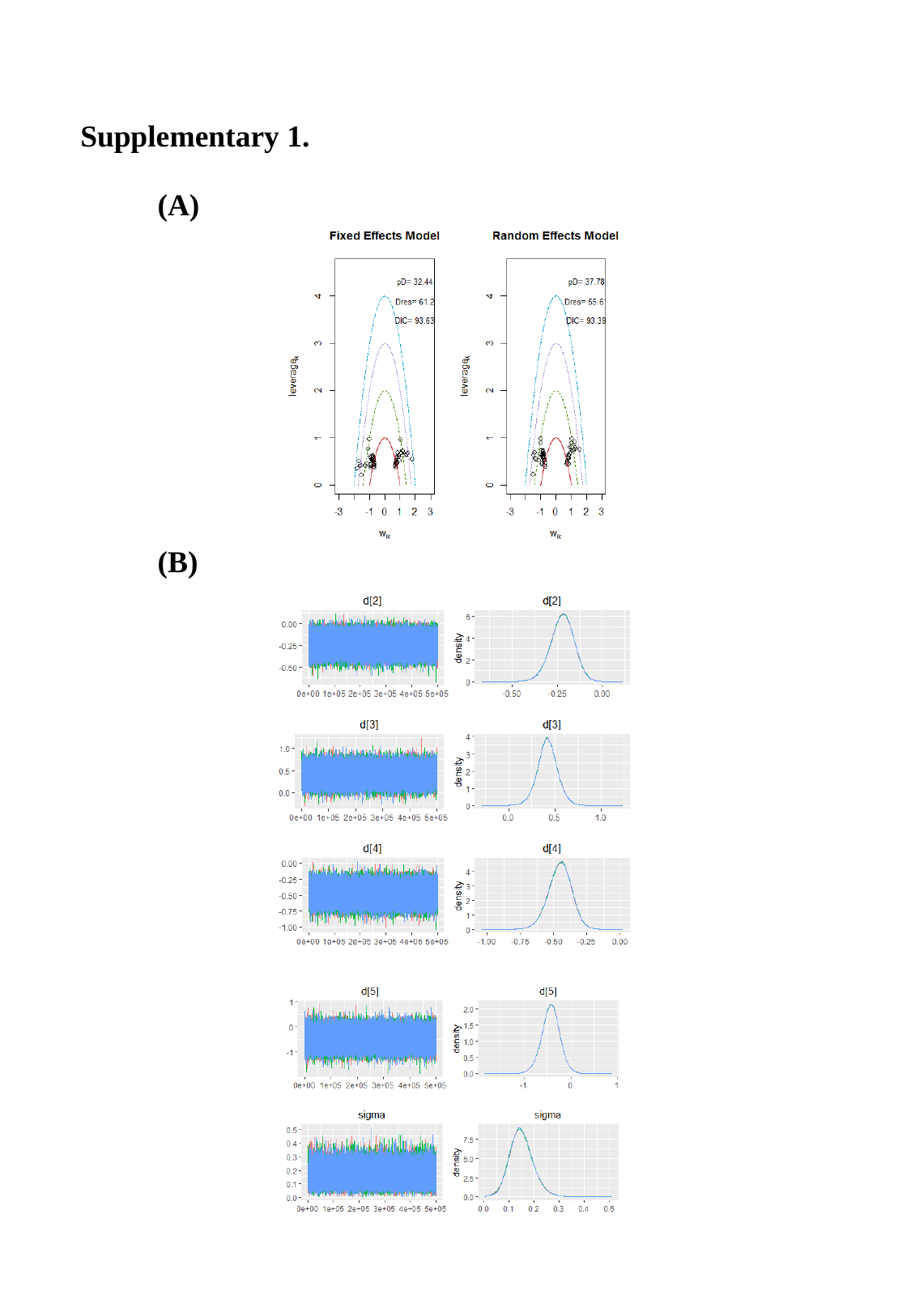

Supplementary 1.
(A)
(B)

Supplement: Multimedia component 1 [file mmc1.pptx]

## Slide 1
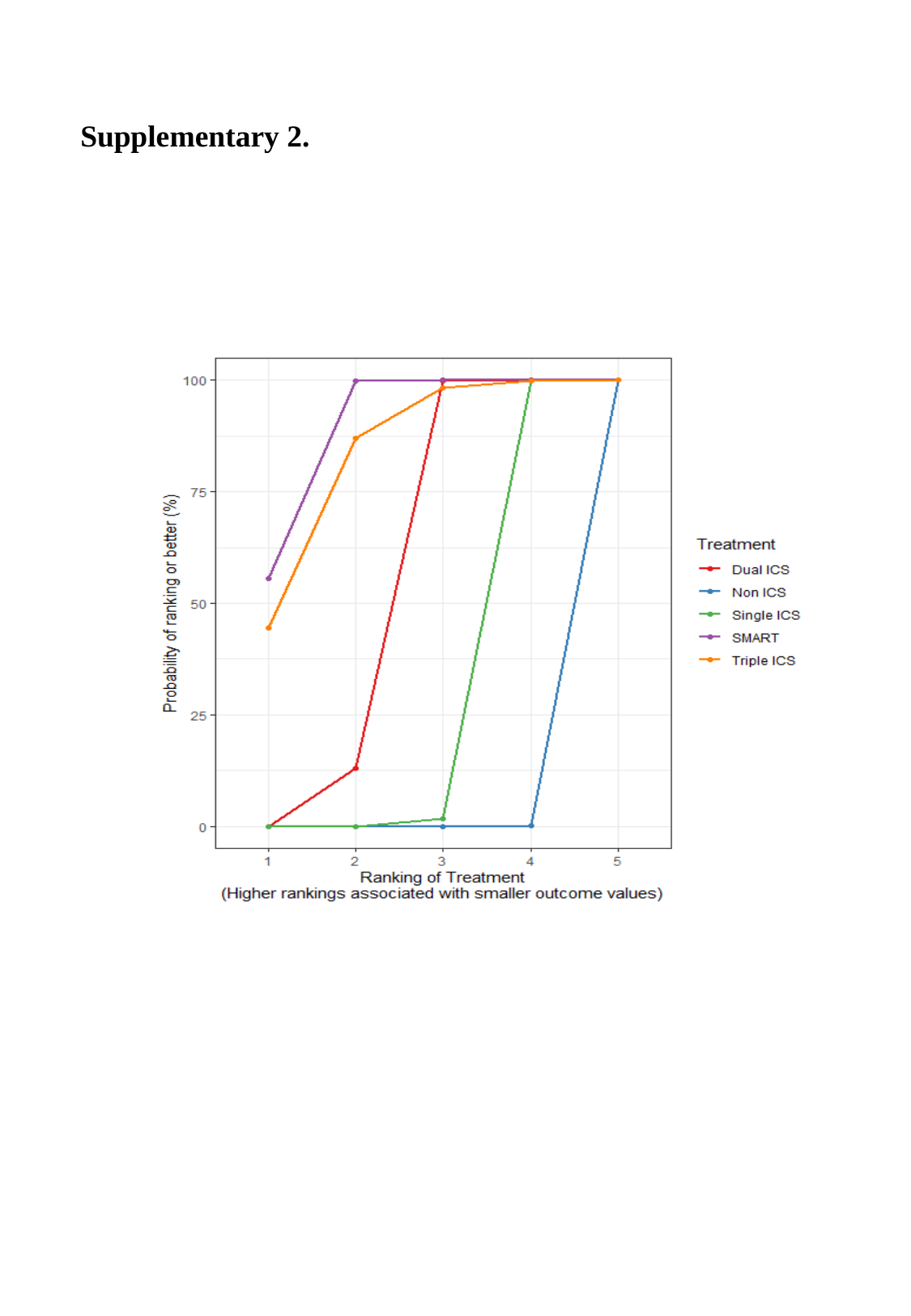

Supplementary 2.

Supplement: Multimedia component 2 [file mmc2.pptx]

## Slide 1
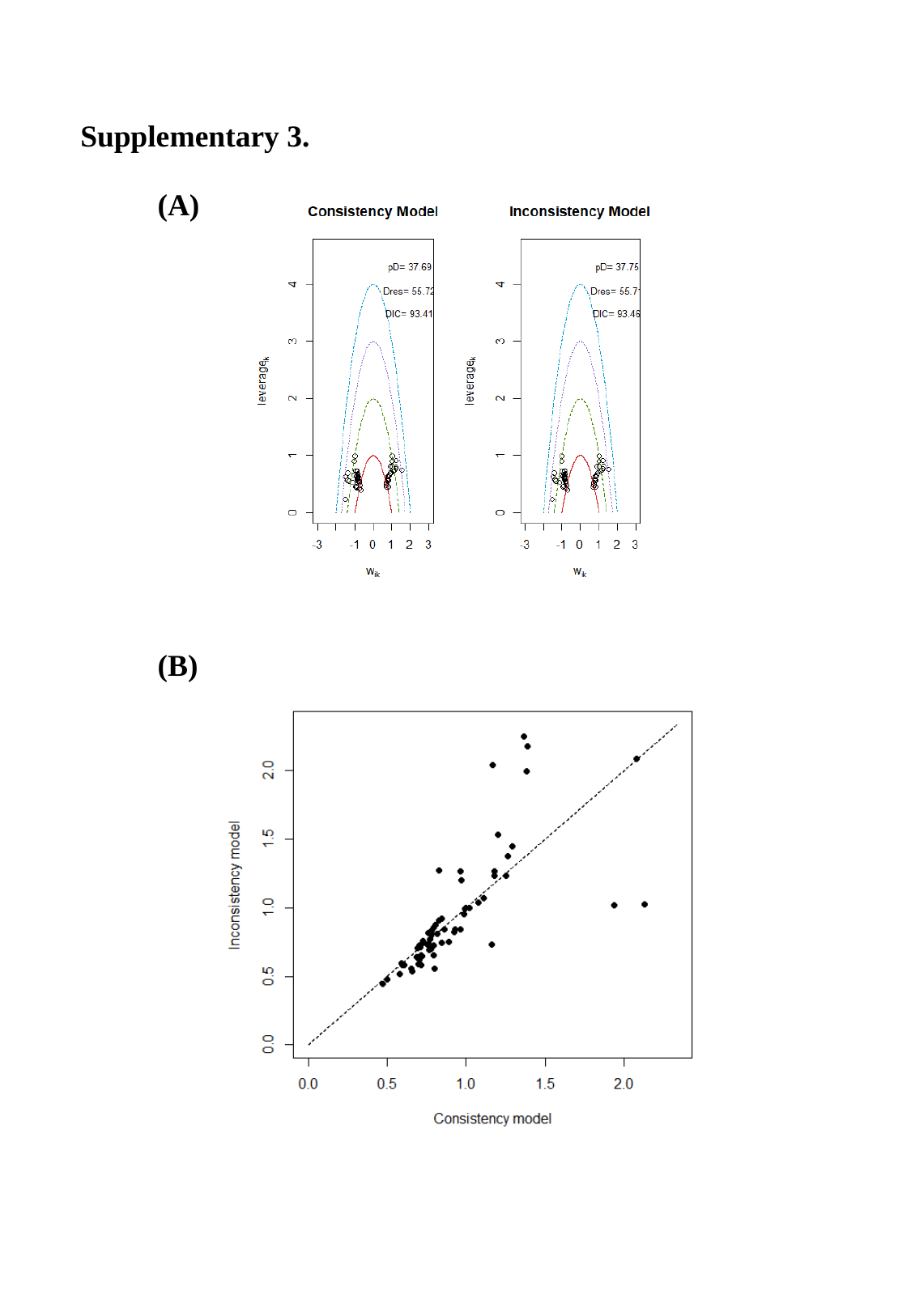

Supplementary 3.
(A)
(B)

Supplement: Multimedia component 3 [file mmc3.pptx]
